# Differential Association of Sex Hormones with Metabolic Parameters and Body Composition in Men and Women from the United States

**DOI:** 10.3390/jcm12144783

**Published:** 2023-07-19

**Authors:** Stefano Ciardullo, Francesca Zerbini, Rosa Cannistraci, Emanuele Muraca, Silvia Perra, Alice Oltolini, Gianluca Perseghin

**Affiliations:** 1 Department of Medicine and Rehabilitation, Policlinico di Monza, 20900 Monza, Italy; francesca.zerbini@policlinicodimonza.it (F.Z.); rosa.cannistraci@policlinicodimonza.it (R.C.); emanuele.muraca@policlinicodimonza.it (E.M.); silvia.perra@policlinicodimonza.it (S.P.); alice.oltolini@policlinicodimonza.it (A.O.); gianluca.perseghin@policlinicodimonza.it (G.P.); 2 Department of Medicine and Surgery, University of Milano Bicocca, 20900 Milan, Italy

**Keywords:** testosterone, estradiol, SHBG, DXA, visceral adipose tissue

## Abstract

Sex hormones impact body composition. Data on the specific impact of each hormone on different body depots in men and women are scarce. The aim of this study is to evaluate the association between testosterone, estradiol and body fat distribution in the general population. This is a population-based cross-sectional study based on data from the 2013–2016 cycles of the National Health and Nutrition Examination Survey. Dual energy X-ray absorptiometry (DXA) and liquid chromatography tandem mass spectrometry were performed on participants aged 18–59 years to evaluate body composition and sex hormone levels, respectively. Weighted multivariable linear regression analyses were performed to evaluate the association between these parameters after adjustment for potential confounders. A total of 6655 participants (3309 males and 3346 females) was included in the analysis. Men with lower testosterone levels were older, had a higher body mass index (BMI) and had a generally unfavorable metabolic profile, while no specific trends were found in women. Among men, testosterone was positively associated with lean body mass and was negatively associated with fat mass and the android/gynoid (A/G) ratio, while an opposite trend was found for estradiol. Among women, testosterone did not impact body composition, while estradiol levels were positively associated with lean mass and were negatively associated with fat mass. Our results support the notion that the impact of different sex hormones on specific fat depots varies substantially between men and women.

## 1. Introduction

Increasing evidence suggests that regional body fat distribution, rather than total body fat mass, might be a critical factor linking obesity and metabolic conditions such as insulin resistance, type 2 diabetes and nonalcoholic fatty liver disease [1,2]. Importantly, sexual dimorphisms in fat distributions have been observed [3]. In general, women have higher body fat percentages than men, who tend to have a higher lean body mass [4,5,6]. Furthermore, men are more likely to accumulate fat in the abdomen in a so-called android or apple-shaped pattern, which is associated with a higher risk of metabolic derangements [7]. On the other hand, females have a more peripheral distribution of fat in early adulthood; nonetheless, greater parity and menopause both induce a more android fat distribution with increasing age [6].

These differences, together with the changes seen after menopause or other altered states of androgen deficiency or excess, suggest a causal role for sex hormones in human body fat distribution patterning [8]. Nonetheless, the role of each specific hormone remains quite elusive, notwithstanding the abundant literature on the topic being produced in the last decades, which is characterized by frequently inconsistent results [9]. Among the reasons for these inconsistencies, the measurement of body fat depots and sex steroid hormones has been crucial. It is well accepted that imaging methods (such as dual X-ray absorptiometry, DXA and magnetic resonance) are superior to anthropometric measurements in assessing fat distribution. Similarly, liquid chromatography–tandem mass spectrometry (LC-MS/MS) is considered superior to immunoassays for the measurement of several sex hormones, especially at low concentrations [10,11].

Another aspect that it is not always considered in similar analyses is the role of race–ethnicity on both body composition and sex hormone levels. Previous studies showed that African American women had more bone and muscle mass but less fat as a percentage of body weight than white women after controlling for ethnic differences in age, body weight and height [12]. Furthermore, racial differences in central adiposity in men are established in early adulthood and influence circulating sex-hormone-binding globulin and thereby total testosterone levels [13]. In particular, significantly higher serum levels of total testosterone and sex-hormone-binding globulin (SHBG) were found in African American men than in Caucasian men, whereas free testosterone levels were similar in both groups.

In the present study, we aim to evaluate the association between sex hormones, body composition and fat distribution in the general United States population. To achieve this goal, we analyzed data from the 2013–2016 cycle of the National Health and Nutrition Examination Survey (NHANES) in which total testosterone and estradiol levels were measured through LC-MS/MS and in which DXA was performed to evaluate body composition.

## 2. Materials and Methods

The current analysis utilized publicly available data obtained from the National Center for Health Statistics.

The data can be accessed at https://wwwn.cdc.gov/nchs/nhanes/default.aspx (accessed on 10 April 2023). The analysis focused on the 2013–2016 cycles of NHANES, which is a comprehensive survey conducted in the United States by the National Center for Health Statistics. NHANES is an ongoing cross-sectional complex survey that aims to include individuals from the general population of all ages who are not institutionalized. In order to achieve this, NHANES employs a stratified, multistage, clustered probability sampling design. The survey deliberately oversamples certain demographic groups, such as non-Hispanic black and Hispanic individuals, those with low income and older adults. The survey process involves a structured interview conducted in the participants’ homes followed by a standardized health examination that includes both physical examinations and laboratory tests. Detailed information on the data collection methodology can be found elsewhere [14,15]. The original NHANES survey received approval from the Centers for Disease Control and Prevention Research Ethics Review Board, and written informed consent was obtained from all adult participants. The present analysis, utilizing a completely deidentified dataset, was determined to be exempt from review by the institutional review board at our institution.

### 2.1. Laboratory and Clinical Data

Participants provided self-reported information on their age, sex, race–ethnicity (categorized as non-Hispanic white, non-Hispanic black, Hispanic, non-Hispanic Asian or other), education, smoking status and previous medical history. During the Mobile Examination Center (MEC) visit, body measurements such as height (cm), weight (kg) and waist circumference (cm) were recorded. Body mass index (BMI) was calculated by dividing weight in kilograms by the square of height in meters. Trained physicians obtained blood pressure measurements using a mercury sphygmomanometer and an appropriately sized cuff. After a 5 min seated resting period, three consecutive auscultatory blood pressure readings were taken. The average of these three measurements represented both systolic and diastolic blood pressure values. Hypertension was defined in accordance with the 2018 European Society of Cardiology (ESC)/European Society of Hypertension (ESH) Guidelines, which considered a systolic blood pressure (SBP) value of ≥140 mmHg and/or a diastolic blood pressure (DBP) value of ≥90 mmHg to be hypertensive, or by the current use of antihypertensive medication [16]. The remaining participants were categorized into three groups based on their blood pressure readings as follows: optimal BP (SBP of <120 mmHg and DBP of <80 mmHg), normal BP (SBP of 120–129 mmHg and/or DBP of 80–84 mmHg) and high normal BP (SBP of 130–149 mmHg and/or DBP of 85–89 mmHg).

Participants were classified as having diabetes if they responded affirmatively to the question “Other than during pregnancy, have you ever been informed by a doctor or health professional that you have diabetes or sugar diabetes?” Additionally, individuals were considered to have diabetes if they met at least two of the following criteria: HbA1c level of ≥6.5%, random plasma glucose level of ≥200 mg/dL or fasting plasma glucose level of ≥126 mg/dL according to the diagnostic criteria recommended by the American Diabetes Association. [17]. Laboratory methods for measurements of HbA1c and glucose are reported in detail elsewhere [18,19]. We evaluated the prevalence of liver steatosis by calculating the fatty liver index (FLI) [20]:

e^y^/(1 + e^y^) × 100 (1)

where y = 0.953 × ln(triglycerides (mg/dL)) + 0.139 × BMI (kg/m^2^) + 0.718 × ln(GGT (U/L)) + 0.053 × waist circumference (cm) − 15.745. A FLI value ≥ 60 was considered to be indicative of liver steatosis as originally proposed [20].

To evaluate the degree of insulin resistance the homeostasis model assessment of insulin resistance based on serum fasting glucose and serum fasting insulin was calculated as originally described [21].

### 2.2. Sex Hormone Measurements

Simultaneous measurements of total testosterone and estradiol in serum were performed using isotope dilution–liquid chromatography–tandem mass spectrometry (ID-LC-MS/MS) method for routine analysis developed by CDC. LC-MS/MS was proposed as the preferable method in the Endocrine Society Position Statement [22]. The method was created for high sample throughput and demonstrates high accuracy and precision over multiple years. SHBG is based on the reaction of SHBG with immunoantibodies and chemiluminescence measurements of the reaction products that occurs after two incubation periods and subjection to a magnetic field. The microparticles are captured on an electrode, where a chemiluminescent reaction occurs and can be measured by a photomultiplier tube. The readings are compared to an instrument- and lot-specific calibration curve. We calculated free testosterone (pmol/L) using the empirical equation suggested by Ly et al. [23], which was derived from a large dataset comprising over 4000 consecutive blood samples in which free testosterone as well as total testosterone and SHBG were measured. It showed good accuracy compared with other proposed equations in subsequent studies [24].

### 2.3. Whole Body DXA

Participants between the ages of 18 and 59 underwent whole-body dual-energy X-ray absorptiometry (DXA) scans, with certain criteria for exclusion. Pregnant women, individuals who reported recent use of radiographic contrast material (barium) within the past 7 days and those with a self-reported weight exceeding 450 pounds or height over 6′5″ were not included due to limitations of the DXA table. The scans were conducted using Hologic Discovery model A densitometers (Hologic, Inc., Bedford, MA, USA) and were analyzed using Hologic APEX software version 4.0. Trained and certified radiology technologists administered the DXA examinations. The Hologic APEX software employed in the scan analysis defined the android and gynoid (A/G) regions. The android area was characterized as the lower trunk region between two lines: the lower side of the pelvic horizontal cut line and an automatically placed line above the pelvic line. The upper gynoid line was positioned 1.5 times the height of the android region below the pelvic line, while the lower gynoid line was positioned to maintain a distance between the two gynoid lines that was twice the height of the android region. These line placements were automatically determined by the Hologic software.

Previous studies have shown that fat deposition in the android region is associated with increased risk of cardiovascular disease, hypertension, hyperlipidemia, insulin resistance and type 2 diabetes [25], while gynoid fat deposition is associated with decreased risk of metabolic and cardiovascular diseases (Folsom, 2000).

Further information on the DXA procedures can be found on the specific manuals on the NHANES website [26].

### 2.4. Statistical Analysis

The statistical analyses were performed utilizing Stata version 16.0 (StataCorp, College Station, TX, USA) and taking into consideration the intricate survey design of NHANES. To account for the recommended guidelines of the NCHS, appropriate weighting was applied to each analysis. Categorical variables were presented as weighted proportions ± standard error (SE), while continuous variables were expressed as weighted means ± SE.

Participants’ characteristics by quartiles of total testosterone concentrations were compared using linear regression for continuous variables and the design-adjusted Rao–Scott chi-square test for categorical variables. Multivariable linear regression analysis was performed in order to evaluate the effect of sex hormones on body composition after adjustment for potential confounders. Analyses were performed separately on men and women. Covariates included in the model were age, race–ethnicity and BMI. A two-tailed value of *p* < 0.05 was considered statistically significant.

### 2.5. Analysis Sample

**Figure 1:**
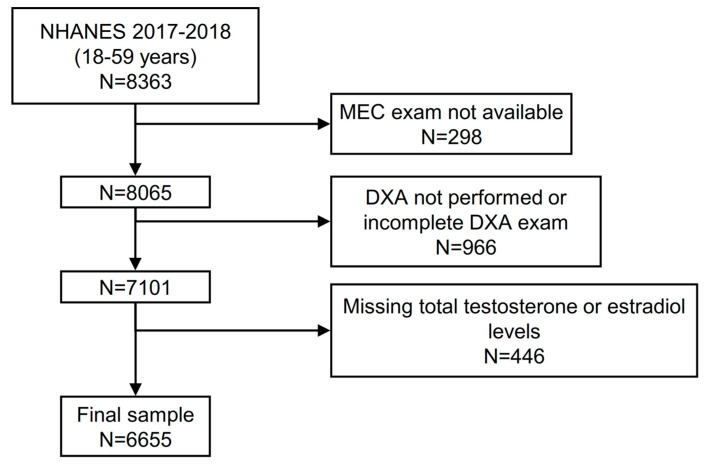
Flow chart of the study participants. Abbreviations: NHANES represents National Health and Nutrition Examination Survey.

Of a total of 8363 participants aged 18–59 years in the 2013–2016 NHANES cycles, 8065 attended a MEC visit. We initially excluded individuals without a complete DXA exam, leading to a population of 7101 participants. Of these, 446 had missing values in their testosterone and/or estradiol levels, leaving a final sample of 6655 participants with complete data (Figure 1).

## 3. Results

### 3.1. Features of Participants According to Testosterone Levels

Table 1 shows the clinical and biochemical characteristics of the male participants stratified by the quartiles of the total testosterone concentrations. In total, 15.8% (95% CI 14.1–17.7) of all the males had a low testosterone level (<264 ng/dL or 9.2 nmol/L) [27].

Lower levels of testosterone were associated with older age; a higher BMI; a lower proportion of non-Hispanic black participants; a lower proportion of smokers; a higher prevalence of diabetes; a fatty liver; and worse cardiometabolic parameters, including higher systolic and diastolic blood pressures, total cholesterol, triglyceride levels and homeostatic model for insulin resistance (HOMA-IR). Compared with the participants in quartiles 2–4, the participants in quartile 1 (testosterone levels < 309 ng/dL) had a higher percent fat, A/G ratio and visceral and subcutaneous adipose tissue volume and a lower percent lean mass. The participants with lower testosterone levels also had lower estradiol concentrations.

Table 2 shows the clinical and biochemical characteristics of the female participants stratified by the quartiles of the total testosterone concentrations. Lower levels of testosterone were associated with older age; a higher proportion of non-Hispanic Asian participants; a higher prevalence of diabetes; a fatty liver; and higher blood pressure, total cholesterol, triglyceride levels and homeostatic model for insulin resistance (HOMA-IR). No difference was found between the BMI and smoking habits. Compared with the participants in quartiles 2–4, the participants in quartile 1 had a higher visceral adipose tissue volume and a lower subcutaneous fat volume along with a lower gynoid fat mass. The participants with lower testosterone levels also had lower estradiol concentrations.

### 3.2. Independent Predictors of Body Composition

To better dissect the relative contribution of testosterone and estradiol to body composition, we performed multivariable linear regression analyses adjusted for age, BMI and race–ethnicity in both men and women.

As shown in Table 3, among the men, testosterone was positively associated with lean body mass and was negatively associated with fat mass and the android/gynoid (A/G) ratio, while an opposite trend was found for estradiol. Moreover, compared with non-Hispanic white individuals, the non-Hispanic black participants had a higher lean mass, a lower fat mass and a lower A/G ratio. An opposite trend was identified for non-Hispanic Asians. As shown in Supplementary Table S1, free testosterone was correlated positively with lean mass and negatively with fat mass, with no significant association with the A/G ratio.

Among the women, testosterone did not impact body composition, while estradiol levels were positively associated with lean mass and were negatively associated with fat mass (Table 4). Moreover, compared with non-Hispanic white individuals, the non-Hispanic black participants had a higher lean mass and a lower fat mass. An opposite trend was again identified for non-Hispanic Asians, who also displayed a higher A/G ratio.

In both sexes, the models showed higher performance in predicting percent lean and fat mass compared with the A/G ratio.

## 4. Discussion

In this large, cross-sectional, population-based study performed on a representative sample of the multiethnic United States population, we investigated the association of sex hormones with metabolic parameters and body composition. We made a series of observations. First, among men, testosterone was strongly and positively associated with lean body mass and was negatively associated with fat mass and the A/G ratio, while an opposite trend was found for estradiol. Second, testosterone levels in women were not associated with body composition after accounting for potential confounders, while estradiol levels were. Third, racial–ethnic differences in body composition were present irrespective of the sex hormone concentrations. Finally, the participants with lower testosterone levels tended to present with lower estradiol concentrations and vice versa.

Our results on the testosterone concentrations in men are in agreement with several cross-sectional and longitudinal studies, showing that obesity as well as an increased accumulation of visceral fat and metabolic syndrome (which is a composite of elevated blood glucose, elevated blood pressure, elevated triglycerides, low HDL cholesterol and elevated waist circumference [28]) are associated with proportionally lower levels of total testosterone [29,30,31,32]. The physiological mechanisms underlying this association appear to be bidirectional. In fact, weight loss has been shown to be associated with an increase in serum testosterone proportional to the amount of weight lost [33]. On the other hand, most randomized controlled trials (though not all [34]) showed that a testosterone treatment led to a significant decrease in fat body mass and an increase in lean body mass [35].

Evidence of the role of testosterone in women is less strong. In a previous population-based study performed in the US, the authors showed that body composition measures (BMI, percent body fat, lean mass and fat mass) were significantly and positively associated with the total testosterone concentrations in a dose–response manner [36]. The results conflicting with those of the present study might be due to the lack of adjustment for the concomitant estradiol concentrations in the previous study as well as a relatively narrow range of testosterone levels in the women in our study. Indeed, being performed in a general population setting, it is conceivable that our study did not capture a high number of women with significantly elevated levels of total testosterone.

It is possible that the effect of testosterone on body composition in women might be achieved by higher concentrations as suggested by a clinical trial that randomized postmenopausal women to estradiol or estradiol + testosterone. Women in the estradiol + testosterone arm achieved an increase in fat-free mass and a reduction in the fat mass/fat-free mass ratio [37]. This topic is especially relevant to women with polycystic ovarian syndrome (PCOS), who also are often afflicted with insulin resistance and steatotic liver disease. Indeed, the current evidence supports a strong link between NAFLD and liver fibrosis and both PCOS and hyperandrogenism in women [38]. Nonetheless, the data are still conflicting on whether this association might be causal or explained by the underlying defects in insulin action that are common to the two conditions [39].

Unfortunately, data were not available in our sample to evaluate whether a history of PCOS might have an impact on body composition independently from androgen levels or the testosterone/estrogen ratio.

Evidence of the association of serum estradiol with body composition in women of child-bearing age is limited. Studies performed on postmenopausal women have shown that estradiol levels are proportional to adipose mass, although they remain in the low range. Importantly, in a previous study performed on 100 healthy postmenopausal women, a higher insulin resistance as well as higher triglyceride, VLDL and IL-6 levels were observed in the participants with higher estrogen levels (i.e., those in the third tertile of the serum estrogen concentrations), but these differences were explained by a concomitant increase in total adiposity [40].

The current study possesses multiple strengths. It relies on data derived from the NHANES cycles, ensuring that the findings can be applied to the entire multiethnic adult population in the United States, specifically those aged 18–59 years. Furthermore, the collection of both biochemical and risk factor data was carried out in a standardized and consistent manner by trained personnel. Lastly, the study utilized two reliable noninvasive methods to assess body fat distribution and the concentrations of sex hormones, both of which exhibited strong performance.

Similarly, it is important to recognize several limitations in this study. Firstly, the cross-sectional design prevents us from establishing a cause–effect relationship between sex hormones and body composition. While there is evidence suggesting a bidirectional cause–effect association, definitive conclusions cannot be drawn. Secondly, although DXA is a valuable tool for assessing body composition, more precise techniques, such as computed tomography and magnetic resonance screening, are available. However, these advanced techniques come with higher costs and longer examination times, making their implementation challenging in large population studies. Thirdly, since DXA scans were not conducted on individuals aged 60 years or older, our findings cannot be extrapolated to this specific age group. Consequently, we were unable to examine whether the relationship between body composition and sex hormones in females might be influenced by menopause, a phase when the prevalence of android fat deposition increases among women. This is also relevant since, during menopause, many hormones apart from estrogens may suffer disbalances, such as thyroid hormones [41]. These hormones influence metabolism and may therefore cause an alteration in fat metabolism and storage [42].

Finally, we did not adjust our analyses for physical activity levels, which might positively impact body composition and help preserve gonadal function.

## 5. Conclusions

In conclusion, this large cross-sectional study shows that associations between sex hormones and body composition differ between men and women. In men, testosterone was strongly and positively associated with lean body mass and was negatively associated with fat mass and the A/G ratio, with an opposite trend found for estradiol; the testosterone levels in women were not associated with body composition after accounting for potential confounders, while estradiol levels were. Our data might inform clinicians of the importance to evaluate both hormone levels to have a more complete picture of patients’ sexual and metabolic health. Given that our results were obtained in an ethnically diverse population, we speculate that similar findings might be documented outside the US, even though large epidemiologic studies are needed.

## Figures and Tables

**Table 1:** Features of male participants according to quartiles of total testosterone concentrations.

	Total Testosterone Quartiles								
	Q1 (<310 ng/dL)		Q2 (<403 ng/dL)		Q3 (<530 ng/dL)		Q4 (≥530 ng/dL)		*p*-Trend
	*n* or Mean	% or SE	*n* or Mean	% or SE	*n* or Mean	% or SE	*n* or Mean	% or SE	
Age (years)	40.6	(0.5)	39.2	(0.5)	38.0	(0.6)	35.6	(0.6)	<0.001
BMI (kg/m^2^)	32.0	(0.3)	29.4	(0.2)	27.6	(0.2)	25.6	(0.2)	<0.001
Diabetes	100	12.1%	57	6.9%	54	6.6%	43	5.2%	<0.001
Race–ethnicity (%)									0.091
Non-Hispanic white	523	63.0%	518	62.8%	526	63.5%	482	58.3%	
Hispanic	156	18.8%	155	18.8%	144	17.4%	148	18.0%	
Non-Hispanic black	72	8.6%	83	10.0%	86	10.4%	113	13.7%	
Non-Hispanic Asian	46	5.5%	45	5.4%	47	5.7%	42	5.1%	
Other	34	4.1%	24	3.0%	25	3.0%	41	5.0%	
Fatty liver index									<0.001
<30	77	9.4%	163	19.9%	277	33.8%	446	54.1%	
30–60	108	13.1%	175	21.4%	219	26.7%	179	21.7%	
>60	635	77.4%	480	58.7%	323	39.5%	199	24.2%	
Cigarette smoker									<0.001
Never	456	54.9%	484	58.7%	459	55.4%	397	48.0%	
Former	223	26.9%	172	20.9%	171	20.6%	173	21.0%	
Current	151	18.2%	169	20.4%	199	24.0%	256	31.0%	
Testosterone (ng/dL)	241.1	(2.2)	356.1	(1.1)	464.3	(1.6)	680.6	(6.2)	<0.001
SHBG (nmol/L)	25.4	(0.4)	32.4	(0.7)	40.0	(0.8)	52.8	(1.0)	<0.001
Free testosterone (pmol/L)	115.4	(1.6)	185.8	(1.3)	245.8	(1.7)	351.6	(4.5)	<0.001
Estradiol (pg/mL)	21.5	(0.4)	22.6	(0.3)	24.9	(0.3)	29.9	(0.5)	<0.001
SBP (mmHg)	124.4	(0.6)	122.4	(0.6)	119.7	(0.6)	119.8	(0.6)	<0.001
DBP (mmHg)	74.2	(0.5)	72.8	(0.4)	71.3	(0.4)	70.3	(0.5)	<0.001
FPG (mg/dL)	115.2	(0.9)	106.9	(0.8)	104.4	(0.8)	102.5	(0.8)	<0.001
Insulin (uU/mL)	20.4	(0.5)	16.9	(0.7)	11.5	(0.5)	8.5	(0.3)	<0.001
HOMA-IR	6.2	(0.2)	4.9	(0.3)	3.1	(0.1)	2.2	(0.1)	<0.001
Total cholesterol (mg/dL)	196.2	(1.8)	191.3	(2.3)	187.6	(1.7)	183.9	(1.7)	<0.001
Triglycerides (mg/dL)	238.7	(7.3)	195.9	(13.9)	149.0	(4.4)	117.6	(5.0)	<0.001
**DXA parameters**									
Trunk percent fat	31.7	(0.3)	29.1	(0.3)	26.2	(0.3)	23.1	(0.3)	<0.001
Lean mass (g)	61,977.8	(433.9)	57,983.8	(384.5)	55,659.1	(327.6)	53,939.1	(417.3)	<0.001
Percent fat	30.9	(0.2)	28.5	(0.2)	26.1	(0.2)	23.7	(0.3)	<0.001
Android fat mass (g)	3276.0	(66.3)	2621.9	(55.1)	2162.6	(57.6)	1710.7	(49.0)	<0.001
Gynoid fat mass (g)	4921.3	(82.0)	4247.6	(60.6)	3792.5	(64.9)	3311.8	(63.6)	<0.001
Android to gynoid ratio	1.2	(0.0)	1.1	(0.0)	1.1	(0.0)	1.0	(0.0)	<0.001
Android percent fat	36.2	(0.3)	33.4	(0.3)	30.1	(0.4)	26.4	(0.4)	<0.001
Gynoid percent fat	31.2	(0.2)	29.7	(0.2)	28.1	(0.2)	26.1	(0.3)	<0.001
Visceral adipose tissue volume	754.0	(14.4)	614.4	(13.4)	529.3	(12.9)	435.9	(12.3)	<0.001
Subcutaneous fat volume	1885.2	(35.3)	1574.5	(30.0)	1293.4	(31.4)	1019.3	(28.3)	<0.001
Percent lean	66.8	(0.2)	69.0	(0.2)	71.2	(0.2)	73.5	(0.3)	<0.001

Data are expressed as weighted proportions (±standard error (SE)) for categorical variables and as weighted means ± SE for continuous variables. Linear regression and Rao–Scott chi-squared test were used to compare groups. In order to reduce the risk of spurious significant results, when comparing each group with all the others (multiple comparison), we preferred to report *p*-values for trends of the evaluated variables across testosterone quartiles. Abbreviations: BMI represents body mass index, T2DM represents type 2 diabetes mellitus and BP represents blood pressure.

**Table 2:** Features of female participants according to quartiles of total testosterone concentrations.

	Total Testosterone Quartiles								
	Q1 (<14.7 ng/dL)		Q2 (<21.1 ng/dL)		Q3 (<29.4 ng/dL)		Q4 (≥29.4 ng/dL)		*p*-Trend
	*n* or Mean	% or SD	*n* or Mean	% or SD	*n* or Mean	% or SD	*n* or Mean	% or SD	
Age (years)	44.7	(0.5)	41.1	(0.5)	37.5	(0.5)	33.8	(0.5)	<0.001
BMI (kg/m^2^)	29.1	(0.3)	29.7	(0.3)	29.3	(0.3)	29.1	(0.3)	0.735
Diabetes	106	12.6%	59	7.1%	47	5.6%	24	2.9%	<0.001
Race–ethnicity									0.208
Non-Hispanic white	496	58.8%	523	62.7%	514	61.5%	519	62.4%	
Hispanic	149	17.7%	149	17.9%	154	18.4%	130	15.6%	
Non-Hispanic black	109	12.9%	89	10.7%	96	11.5%	106	12.7%	
Non-Hispanic Asian	60	7.2%	47	5.6%	48	5.8%	37	4.4%	
Other	29	3.4%	26	3.1%	24	2.9%	40	4.8%	
Fatty liver index									0.019
<30	309	37.5%	344	41.6%	374	45.1%	406	49.6%	
30–60	160	19.5%	141	17.1%	141	17.0%	135	16.5%	
>60	355	43.1%	340	41.2%	315	37.9%	278	33.9%	
Cigarette smoker									0.007
Never	557	66.1%	545	65.3%	558	66.7%	507	60.9%	
Former	138	16.4%	146	17.5%	120	14.4%	102	12.3%	
Current	148	17.5%	143	17.2%	158	18.9%	223	26.8%	
Testosterone (ng/dL)	10.8	(0.1)	18.0	(0.1)	24.8	(0.1)	47.4	(2.2)	<0.001
SHBG (nmol/L)	62.6	(2.0)	74.2	(2.6)	78.2	(2.5)	88.0	(2.8)	<0.001
Free testosterone (pmol/L)	1.9	(0.1)	5.1	(0.1)	7.9	(0.1)	15.0	(0.7)	<0.001
Estradiol (pg/mL)	36.1	(2.1)	62.7	(3.2)	88.6	(3.9)	113.4	(5.7)	<0.001
SBP (mmHg)	119.2	(0.7)	116.9	(0.6)	114.6	(0.6)	113.8	(0.6)	<0.001
DBP (mmHg)	72.0	(0.4)	70.3	(0.4)	69.2	(0.4)	68.1	(0.4)	<0.001
FPG (mg/dL)	110.4	(1.2)	101.8	(0.6)	100.5	(0.7)	95.7	(0.4)	<0.001
Insulin (uU/mL)	12.7	(0.3)	12.4	(0.3)	11.6	(0.4)	10.8	(0.2)	0.040
HOMA-IR	3.8	(0.1)	3.3	(0.1)	3.1	(0.2)	2.6	(0.1)	0.009
Total cholesterol (mg/dL)	195.0	(2.2)	193.6	(1.6)	184.8	(1.8)	187.7	(1.7)	0.002
Triglycerides (mg/dL)	153.8	(4.7)	131.4	(3.7)	122.3	(6.7)	112.7	(3.4)	<0.001
**DXA parameters**									
Trunk percent fat	36.8	(0.3)	37.1	(0.3)	36.2	(0.4)	35.8	(0.3)	0.019
Lean mass (g)	40,828.0	(356.6)	41,641.4	(347.0)	41,463.6	(346.4)	41,434.4	(327.9)	0.195
Percent fat	39.5	(0.3)	40.1	(0.3)	39.4	(0.3)	39.2	(0.3)	0.228
Android fat mass (g)	2529.7	(62.4)	2608.0	(58.7)	2502.6	(62.2)	2491.6	(59.2)	0.419
Gynoid fat mass (g)	5206.1	(83.0)	5543.4	(85.9)	5559.4	(87.9)	5523.8	(83.7)	0.018
Android to gynoid ratio	0.9	(0.0)	0.9	(0.0)	0.9	(0.0)	0.9	(0.0)	0.040
Android percent fat	38.4	(0.4)	39.3	(0.4)	38.4	(0.4)	38.4	(0.4)	0.614
Gynoid percent fat	41.8	(0.2)	42.7	(0.2)	42.5	(0.2)	42.3	(0.2)	0.393
Visceral adipose tissue volume	574.0	(15.8)	543.2	(14.9)	489.9	(14.4)	456.3	(12.5)	<0.001
Subcutaneous fat volume	2055.2	(36.3)	2158.7	(36.3)	2111.4	(39.3)	2134.0	(38.8)	0.331
Percent lean	58.2	(0.3)	57.6	(0.2)	58.3	(0.3)	58.5	(0.3)	0.272

Data are expressed as weighted proportions (±standard error (SE)) for categorical variables and as weighted means ± SE for continuous variables. Linear regression and Rao–Scott chi-squared test were used to compare groups. In order to reduce the risk of spurious significant results, when comparing each group with all the others (multiple comparison), we preferred to report *p*-values for trends of the evaluated variables across testosterone quartiles. Abbreviations: BMI represents body mass index, T2DM represents type 2 diabetes mellitus and BP represents blood pressure.

**Table 3:** Multivariable linear regression model assessing the contribution of sex hormones to body composition in male participants.

	Lean Mass (%)			Fat Mass (%)			Android/Gynoid Ratio		
	B	95% CI	*p*-Value	B	95% CI	*p*-Value	B	95% CI	*p*-Value
Age (years)	−0.04	−0.05–−0.02	<0.001	0.04	0.02–0.06	<0.001	0.00	0.00–0.01	<0.001
BMI (kg/m^2^)	−0.65	−0.70–−0.61	<0.001	0.71	0.67–0.76	<0.001	0.01	0.01–0.01	<0.001
Race–ethnicity									
Non-Hispanic white	Ref			Ref			Ref		
Hispanic	0.03	−0.34–0.40	0.871	−0.01	−0.41–0.39	0.969	0.05	0.03–0.06	<0.001
Non-Hispanic black	2.77	2.30–3.24	<0.001	−3.00	−3.48–−2.52	<0.001	−0.03	−0.05–−0.01	0.005
Non-Hispanic Asian	−1.26	−1.84–−0.68	<0.001	1.36	0.76–1.95	<0.001	0.07	0.04–0.09	<0.001
Other	0.73	−0.19–1.65	0.118	−0.79	−1.76–0.17	0.102	−0.02	−0.05–0.01	0.185
Testosterone (ng/dL)	0.01	0.01–0.01	<0.001	−0.01	−0.01–−0.01	<0.001	−0.00	−0.00–−0.00	<0.001
Estradiol (pg/mL)	−0.06	−0.09–−0.04	<0.001	0.06	0.03–0.09	<0.001	0.00	0.00–0.00	0.003
R^2^	0.59			0.62			0.38		

Abbreviations: CI represents confidence interval, BMI represents body mass index and A/G ratio represents android/gynoid ratio.

**Table 4:** Multivariable linear regression model assessing the contribution of sex hormones to body composition in female participants.

	Lean Mass (%)			Fat Mass (%)			Android/Gynoid Ratio		
	B	95% CI	*p*-Value	B	95% CI	*p*-Value	B	95% CI	*p*-Value
Age (years)	−0.05	−0.07–−0.04	<0.001	0.06	0.04–0.08	<0.001	0.00	0.00–0.00	0.003
BMI (kg/m^2^)	−0.62	−0.65–−0.59	<0.001	0.68	0.65–0.71	<0.001	0.01	0.01–0.01	<0.001
Race–ethnicity									
Non-Hispanic white	Ref			Ref			Ref		
Hispanic	−0.63	−1.15–−0.10	0.021	0.70	0.16–1.25	0.012	0.04	0.03–0.05	<0.001
Non-Hispanic black	0.97	0.47–1.46	<0.001	−1.07	−1.58–−0.56	<0.001	−0.01	−0.02–0.01	0.316
Non-Hispanic Asian	−1.00	−1.56–−0.43	0.001	1.07	0.48–1.65	0.001	0.07	0.05–0.09	<0.001
Other	0.57	−0.42–1.57	0.250	−0.61	−1.65–0.44	0.244	0.02	−0.00–0.05	0.050
Testosterone, total (ng/dL)	0.00	−0.00–0.01	0.369	−0.00	−0.01–0.01	0.408	0.00	−0.00–0.00	0.486
Estradiol (pg/mL)	0.00	0.00–0.01	0.003	−0.00	−0.01–−0.00	0.001	−0.00	−0.00–0.00	0.183
R^2^	0.59			0.62			0.39		

Abbreviations: CI represents confidence interval, BMI represents body mass index and A/G ratio represents android/gynoid ratio.

## Data Availability

All data analyzed during this study are publicly available on the NHANES website.

## Supplementary Materials

The following supporting information can be downloaded at: https://www.mdpi.com/article/10.3390/jcm12144783/s1, Table S1: Multivariable linear regression model assessing the contribution of sex hormones on body composition in male participants.

## References

[1] Ciardullo S., Oltolini A., Cannistraci R., Muraca E., Perseghin G. (2022). Sex-related association of NAFLD and liver fibrosis with body fat distribution in the general US population. Am. J. Clin. Nutr..

[2] Ciardullo S., Perseghin G. (2022). Prevalence of elevated liver stiffness in patients with type 1 and type 2 diabetes: A systematic review and meta-analysis. Diabetes Res. Clin. Pract..

[3] Veilleux A., Tchernof A. (2012). Sex differences in body fat distribution. Adipose Tissue Biology.

[4] Lemieux S., Prud’Homme D., Bouchard C., Tremblay A.A., Després J.P. (1993). Sex differences in the relation of visceral adipose tissue accumulation to total body fatness. Am. J. Clin. Nutr..

[5] Van Loan M. Total Body Composition: Birth to Old Age. Human Body Composition 1996. https://us.humankinetics.com/products/human-body-composition-2nd-edition?_pos=1&_sid=c5ddbda9d&_ss=r.

[6] Wells J.C. (2007). Sexual dimorphism of body composition. Best Pract. Res. Clin. Endocrinol. Metab..

[7] Kang S.M., Yoon J.W., Ahn H.Y., Kim S.Y., Lee K.H., Shin H., Choi S.H., Park K.S., Jang H.C., Lim S. (2011). Android Fat Depot Is More Closely Associated with Metabolic Syndrome than Abdominal Visceral Fat in Elderly People. PLoS ONE.

[8] Blouin K., Boivin A., Tchernof A. (2008). Androgens and body fat distribution. J. Steroid Biochem. Mol. Biol..

[9] Tchernof A., Brochu D., Maltais-Payette I., Mansour M.F., Marchand G.B., Carreau A., Kapeluto J. (2011). Androgens and the Regulation of Adiposity and Body Fat Distribution in Humans. Compr. Physiol..

[10] Taylor A.E., Keevil B., Huhtaniemi I.T. (2015). Mass spectrometry and immunoassay: How to measure steroid hormones today and tomorrow. Eur. J. Endocrinol..

[11] Büttler R.M., Martens F., Fanelli F., Pham H.T., Kushnir M.M., Janssen M.J., Owen L., Taylor A.E., Soeborg T., Blankenstein M.A. (2015). Comparison of 7 Published LC-MS/MS Methods for the Simultaneous Measurement of Testosterone, Androstenedione, and Dehydroepiandrosterone in Serum. Clin. Chem..

[12] Gasperino J. (1996). Ethnic differences in body composition and their relation to health and disease in women. Ethn. Health.

[13] Winters S.J., Brufsky A., Weissfeld J., Trump D.L., Dyky M.A., Hadeed V. (2001). Testosterone, sex hormone-binding globulin, and body composition in young adult African American and Caucasian men. Metabolism.

[14] Centers for Disease Control and Prevention 2017: National Health and Nutrition Examination Survey (NHANES). U.S. Department of Health and Human Services. https://wwwn.cdc.gov/nchs/nhanes/continuousnhanes/default.aspx?BeginYear=2017.

[15] Ciardullo S., Muraca E., Zerbini F., Manzoni G., Perseghin G. (2021). NAFLD and Liver Fibrosis Are Not Associated with Reduced Femoral Bone Mineral Density in the General US Population. J. Clin. Endocrinol. Metab..

[16] Williams B., Mancia G., Spiering W., Agabiti Rosei E., Azizi M., Burnier M., Clement D.L., Coca A., De Simone G., Dominiczak A. (2018). 2018 ESC/ESH Guidelines for the management of arterial hypertension: The Task Force for the management of arterial hypertension of the European Society of Cardiology (ESC) and the European Society of Hypertension (ESH). J. Hypertens..

[17] American Diabetes Association (2020). 2. Classification and Diagnosis of Diabetes: Standards of Medical Care in Diabetes—2020. Diabetes Care.

[18] Centers for Disease Control and Prevention 2017: National Health and Nutrition Examination Survey (NHANES). U.S. Department of Health and Human Services. https://wwwn.cdc.gov/nchs/data/nhanes/2017-2018/manuals/2017_MEC_Laboratory_Procedures_Manual.pdf.

[19] Ciardullo S., Perseghin G. (2021). Statin use is associated with lower prevalence of advanced liver fibrosis in patients with type 2 diabetes. Metabolism.

[20] Bedogni G., Bellentani S., Miglioli L., Masutti F., Passalacqua M., Castiglione A., Tiribelli C. (2006). The Fatty Liver Index: A simple and accurate predictor of hepatic steatosis in the general population. BMC Gastroenterol..

[21] Matthews D.R., Hosker J.P., Rudenski A.S., Naylor B.A., Treacher D.F., Turner R.C. (1985). Homeostasis model assessment: Insulin resistance and β-cell function from fasting plasma glucose and insulin concentrations in man. Diabetologia.

[22] Rosner W., Auchus R.J., Azziz R., Sluss P.M., Raff H. (2006). Utility, Limitations, and Pitfalls in Measuring Testosterone: An Endocrine Society Position Statement. J. Clin. Endocrinol. Metab..

[23] Ly L.P., Handelsman D.J. (2005). Empirical estimation of free testosterone from testosterone and sex hormone-binding globulin immunoassays. Eur. J. Endocrinol..

[24] Ly L.P., Sartorius G., Hull L., Leung A., Swerdloff R.S., Wang C., Handelsman D.J. (2010). ORIGINAL ARTICLE: Accuracy of calculated free testosterone formulae in men. Clin. Endocrinol..

[25] Kissebah A.H., Krakower G.R. (1994). Regional adiposity and morbidity. Physiol. Rev..

[26] Shepherd J.A., Fan B., Lu Y., Wu X.P., Wacker W.K., Ergun D.L., Levine M.A. (2012). A multinational study to develop universal standardization of whole-body bone density and composition using GE Healthcare Lunar and Hologic DXA systems. J. Bone Miner. Res..

[27] Travison T.G., Vesper H.W., Orwoll E., Wu F., Kaufman J.M., Wang Y., Lapauw B., Fiers T., Matsumoto A.M., Bhasin S. (2017). Harmonized Reference Ranges for Circulating Testosterone Levels in Men of Four Cohort Studies in the United States and Europe. J. Clin. Endocrinol. Metab..

[28] Grundy S.M., Brewer H.B., Cleeman J.I., Smith S.C., Lenfant C. (2004). Definition of Metabolic Syndrome. Arter. Thromb. Vasc. Biol..

[29] Blouin K., Després J.-P., Couillard C., Tremblay A., Prud’Homme D., Bouchard C., Tchernof A. (2005). Contribution of age and declining androgen levels to features of the metabolic syndrome in men. Metabolism.

[30] Gapstur S.M., Gann P.H., Kopp P., Colangelo L., Longcope C., Liu K. (2002). Serum androgen concentrations in young men: A longitudinal analysis of associations with age, obesity, and race. The CARDIA male hormone study. Cancer Epidemiol. Biomark. Prev..

[31] Laaksonen D.E., Niskanen L., Punnonen K., Nyyssönen K., Tuomainen T.-P., Valkonen V.-P., Salonen R., Salonen J.T. (2004). Testosterone and Sex Hormone–Binding Globulin Predict the Metabolic Syndrome and Diabetes in Middle-Aged Men. Diabetes Care.

[32] Pasquali R., Casimirri F., Cantobelli S., Melchionda N., Labate A.M.M., Fabbri R., Capelli M., Bortoluzzi L. (1991). Effect of obesity and body fat distribution on sex hormones and insulin in men. Metabolism.

[33] Grossmann M., Fui M.T., Dupuis P. (2014). Lowered testosterone in male obesity: Mechanisms, morbidity and management. Asian J. Androl..

[34] Wang C., Eyre D.R., Clark R., Kleinberg D., Newman C., Iranmanesh A., Veldhuis J., Dudley R.E., Berman N., Davidson T. (1996). Sublingual testosterone replacement improves muscle mass and strength, decreases bone resorption, and increases bone formation markers in hypogonadal men—A clinical research center study. J. Clin. Endocrinol. Metab..

[35] Corona G., Giagulli V.A., Maseroli E., Vignozzi L., Aversa A., Zitzmann M., Saad F., Mannucci E., Maggi M. (2016). Therapy of Endocrine Disease: Testosterone supplementation and body composition: Results from a meta-analysis study. Eur. J. Endocrinol..

[36] Sowers M., Beebe J., McConnell D., Randolph J., Jannausch M. (2001). Testosterone concentrations in women aged 25–50 years: Associations with lifestyle, body composition, and ovarian status. Am. J. Epidemiol..

[37] Davis S.R., Walker K.Z., Strauss B.J.G. (2000). Effects of estradiol with and without testosterone on body composition and relationships with lipids in postmenopausal women. Menopause.

[38] Rađenović S.S., Pupovac M., Andjić M., Bila J., Srećković S., Gudović A., Dragaš B., Radunović N. (2022). Prevalence, Risk Factors, and Pathophysiology of Nonalcoholic Fatty Liver Disease (NAFLD) in Women with Polycystic Ovary Syndrome (PCOS). Biomedicines.

[39] Arefhosseini S., Ebrahimi-Mameghani M., Najafipour F., Tutunchi H. (2022). Non-alcoholic fatty liver disease across endocrinopathies: Interaction with sex hormones. Front. Endocrinol..

[40] Marchand G.B., Carreau A.-M., Weisnagel S.J., Bergeron J., Labrie F., Lemieux S., Tchernof A. (2018). Increased body fat mass explains the positive association between circulating estradiol and insulin resistance in postmenopausal women. Am. J. Physiol. Metab..

[41] Del Ghianda S., Tonacchera M., Vitti P. (2014). Thyroid and menopause. Climacteric.

[42] Marlatt K.L., Redman L.M., Beyl R.A., Smith S.R., Champagne C.M., Yi F., Lovejoy J.C. (2020). Racial differences in body composition and cardiometabolic risk during the menopause transition: A prospective, observational cohort study. Am. J. Obstet. Gynecol..

