# Supplementary material for: Differential Association of Sex Hormones with Metabolic Parameters and Body Composition in Men and Women from the United States
Source: J Clin Med. 2023 Jul 19;12(14):4783. doi: 10.3390/jcm12144783 (PMC10381414; doi:10.3390/jcm12144783)
Supplement: Supplementary file 1 [file jcm-12-04783-s001.zip › jcm-2475910-supplementary.pdf]

Supplementary Table S1. Multivariable linear regression model assessing the contribution of sex hormones on body composition in male participants.

	Lean mass (%)			Fat mass (%)			Android/Gynoid Ratio		
	**B**	**95% CI**	***p*-Value**	**B**	**95% CI**	***p*-Value**	**B**	**95% CI**	***p*-Value**
Age (years)	-0.03	-0.05 - -0.01	0.003	0.03	0.01 - 0.05	0.002	0.00	0.00 - 0.01	<0.001
BMI (kg/m^2)	-0.68	-0.73 - -0.63	<0.001	0.74	0.69 - 0.79	<0.001	0.01	0.01 - 0.02	<0.001
Race-Ethnicity									
Non-Hispanic White									
Hispanic	-0.05	-0.47 - 0.37	0.806	0.07	-0.37 - 0.52	0.736	0.04	0.03 - 0.06	<0.001
Non-Hispanic Black	2.87	2.37 - 3.37	<0.001	-3.10	-3.62 - -2.59	<0.001	-0.04	-0.06 - -0.01	0.002
Non-Hispanic Asians	-1.50	-2.10 - -0.91	<0.001	1.61	1.00 - 2.21	<0.001	0.07	0.04 - 0.09	<0.001
Other	0.70	-0.21 - 1.62	0.128	-0.77	-1.74 - 0.19	0.113	-0.02	-0.05 - 0.01	0.202
Free testosterone (pmol/l)	0.01	0.01 - 0.01	<0.001	-0.01	-0.01 - -0.01	<0.001	-0.00	-0.00 - 0.00	0.271
Estradiol (pg/mL)	-0.06	-0.09 - -0.03	<0.001	0.06	0.03 - 0.09	0.001	0.00	-0.00 - 0.00	0.919

Abbreviations: CI, confidence interval; BMI, body mass index; A/G ratio, android/gynoid ratio.
